# Comparative Analysis of Aroma Emissions in ‘Gala’ Apples Stored in Ethanol- and Hexanal-Enriched Controlled Atmosphere

**DOI:** 10.3390/foods14060930

**Published:** 2025-03-09

**Authors:** Erika Jesenko, Rajko Vidrih, Emil Zlatić

**Affiliations:** Department of Food Science and Technology, Biotechnical Faculty, University of Ljubljana, Jamnikarjeva 101, SI-1000 Ljubljana, Slovenia; rajko.vidrih@bf.uni-lj.si (R.V.); emil.zlatic@bf.uni-lj.si (E.Z.)

**Keywords:** shelf life, apple aroma, volatile precursor, long-term storage, ethanol, hexanal, hexyl acetate, 2-methylbutyl acetate

## Abstract

The objectives of this study were to investigate the effects of extended and constant ethanol and hexanal exposure on ‘Gala’ apples’ production of aroma compounds after long-term CA storage. ‘Gala’ apples were stored in a CA under 2 kPa O_2_ and 98 kPa N2 at 1.0 ± 0.1 °C with a constant ethanol (CA-et) or hexanal (CA-he) concentration maintained at 50 µgL^−1^ throughout a six-month storage period. A total of 25 volatile compounds (VOCs) were identified. The odor activity value (OAV) results show that nine VOCs were key aroma compounds. Among them, hexyl acetate, 2-methylbutyl acetate, and 1-butanol were the highest. Hexanal increased the production of hexyl acetate, while ethanol increased the production of 2-methylbutyl acetate and ethyl 2-methylbutanoate. Both precursors promoted the production of 1-butanol after two months of storage and 1 day of shelf life. Overall, the impact of the precursors on aroma production was more pronounced after two months than after six months of storage. Different storage atmospheres significantly influenced VOC correlations, suggesting that ethanol and hexanal addition altered aroma biosynthesis pathways in the ‘Gala’ apples. For varieties like ‘Gala’ that rapidly lose their aroma during CA storage, CA-et and CA-he treatments may be beneficial for short-term storage, enhancing key aroma compounds and improving sensory quality.

## 1. Introduction

Apples (*Malus domestica Borkh*.) are an important fruit crop, with a global production of 93.1 million tons, as reported by the Food and Agriculture Organization of the United Nations in 2022 [1]. This enormous production underlines their importance and widespread consumption worldwide.

In addition to texture and taste, flavor is one of the most critical quality parameters of fruit, significantly influencing consumer acceptance. The aroma profile of fruit plays a key role in determining its sensory characteristics, directly contributing to both smell and taste perception [2,3].

Although more than 350 volatile aroma compounds (VOCs) have been identified in apples, including alcohols, aldehydes, acids, ketones, terpenoids, and esters, a subset of only 20–30 compounds contribute significantly to the characteristic apple aroma [4]. Among these, esters—especially those with an even number of carbon atoms, such as combinations of acetic, butanoic, and hexanoic acids with ethyl, butyl, and hexyl alcohols—dominate the aromatic profile of apples [2,5]. The enzyme alcohol acyltransferase (AAT) is responsible for the final step of ester biosynthesis, which catalyzes the transfer of an acyl group from coenzyme A to an alcohol acceptor [6]. The diversity of esters produced by the action of AAT enzymes is influenced by the availability of substrates, the specificity of the enzyme, and the diversity of genes encoding AAT enzymes [4]. Studies show that fruit aroma diversity is more strongly influenced by substrate availability than by the substrate preference of AAT enzymes, which can utilize a wide range of substrates, including butanol, hexanol, and 2-methylbutanol [7]. Due to the increasing market demand for fruit outside of the growing season and the complexity of supply chains, large quantities of fruit are stored in controlled atmosphere (CA) storage facilities. These storage systems can maintain and control a low oxygen content at a low temperature, which inhibits the ripening process and extends the shelf life of the apples. However, the limited amount of oxygen affects precursor availability and enzyme activity, resulting in the loss of the fruit’s characteristic aromatic compounds [4,8].

Volatile aroma precursors play a key role in regulating and maintaining the aromatic compound metabolism in apples. Adding volatile precursors enables the free diffusion of molecules into the fruit, where aromatic compound metabolism and synthesis take place. Both hexanal and ethanol are known to extend the shelf life and delay the ripening of apples [9,10]. In addition, they can serve as precursors for the synthesis of hexyl and ethyl esters, which play an important role in improving the aroma of apples [4]. Storing apples in an ethanol-saturated atmosphere in a sealed jar for 24 h at room temperature has been shown to increase the synthesis of ethyl esters by threefold [11]. Ethanol is metabolized in fruit tissue in two ways: it can be converted to acetaldehyde under the influence of the enzyme alcohol dehydrogenase (ADH) or esterified by the enzyme AAT [12]. Studies have shown that the addition of hexanal can extend the shelf life of various fruits, including bananas, apples, apricots, and mangoes [9,13,14,15]. In addition, in apples, hexanal is converted to esters, which are important for apple aroma [16,17,18].

Understanding the factors that affect aroma regeneration in apples after cold storage and during shelf life is essential for improving post-harvest fruit quality. Previous studies have primarily focused on the impact of the addition of single volatile precursors at high concentrations on the formation of VOCs in apples. These studies provide valuable insights into the mechanisms of aroma production, but they may not accurately reflect the natural processes that occur in ‘Gala’ apples during cold storage and shelf life. There is limited research on how prolonged exposure to lower concentrations of volatile precursors, such as ethanol and hexanal, influences the metabolic pathways and volatile aroma compound profiles in apples during long-term controlled atmosphere storage. Further research is needed to address this gap and investigate the effects of gradual, low-concentration precursor additions on VOC formation and regeneration. Exposing apples to volatile precursors after cold storage helps to regenerate aroma compounds. Gradual, low-concentration precursor additions may be more effective than single, high-concentration treatments. Investigating the effects of sustained volatile precursor exposure at lower concentrations provides insights into aroma compound synthesis optimization, allowing the fruit’s metabolic pathways to adapt over time.

This study investigates the impact of extended ethanol and hexanal exposure at lower concentrations on the production of aroma compounds in ‘Gala’ apples after long-term CA storage. Both ethanol and hexanal are known to affect fruit ripening, so this study aims to test the precursors’ effects on ethylene production, respiration rate, and color development. Since apple aroma is influenced not only by volatile organic compound (VOC) quantity but also by odor threshold (OT), this study assesses ethanol and hexanal’s impact on the aroma potency of ‘Gala’ apples.

## 2. Materials and Methods

### 2.1. Plant Material and Storage Techniques

Apple fruits, (*Malus domestica Borkh.*) cv. ‘Gala’, were harvested at the commercial maturity stage in the commercial orchard of Sadjarstvo Mirosan (46°13′46″ N, 15°11′05″ E; 248 m above sea level). In total, 140 kg of apples with uniform color and size, free from disease and insect infestation, were selected for this study. Further, 28 kg of randomly selected apples were stored in three separate chambers, each with a volume of 125 L, under controlled atmosphere (CA) conditions with 2 kPa O_2_ and 98 kPa N2 at 1.0 ± 0.1 °C. The CA storage chambers’ relative humidity was manually monitored using a humidity sensor and maintained at 94 ± 2% by means of a saturated calcium chloride solution (0.15 kg per chamber), which absorbed excess moisture. Additionally, the control group comprised 28 kg of apples stored in a normal air atmosphere (NA) at 1 °C.

The chambers were designated as CA, CA-ethanol (CA-et), and CA-hexanal (CA-he). Hexanal or ethanol was added to two of the chambers, maintaining concentrations of 50 µgL^−1^ within the chamber atmosphere throughout the storage period. Six glass vials, each equipped with an 8 mm diameter, 200 μm thick, 3D-printed poly(lactic) (PLA) membrane, were positioned in each storage chamber to deliver the volatile precursors. This maintained steady hexanal or ethanol concentrations of 50 µg kg^−1^ fruit in the chambers throughout the storage duration. This concentration was selected because it exceeds the endogenous concentration and does not damage apple tissue. The apples were stored under these conditions for 6 months.

### 2.2. Ethylene Production and Respiratory Rate

The respiration and ethylene production rates of the fruits used for VOC analysis were determined after 1d and 7d of shelf life at 20 °C following 2 and 6 months of storage. Fruits from each replicate were placed in airtight jars (0.5 L) at 20 °C and continuously flushed with clean air (20 °C). The CO_2_ released from each jar was measured using a CO_2_ infrared analyzer (SprintIR^®^-6S, Gas Sensing Solutions, Glasgow, UK), with results expressed as μg kg^−1^s^−1^.

Ethylene production rates were measured by extracting 1 mL air samples from a 0.5 L sealed glass container. Each jar contained one apple purged with clean air at 20 °C and a flow rate of 30 mL/min for 30 min. The samples were extracted using a 1 mL syringe. The collected air samples were injected into a gas chromatograph (Agilent Technologies 6890 N) fitted with a carbon plot column (60 m × 0.32 mm × 1.5 µm) and a flame ionization detector (FID). The column temperature was maintained at 60 °C and the injector and detector temperatures were 250 °C. The ethylene production rate is expressed as µL kg^−1^ h^−1^.

### 2.3. Fruit Color

Color measurements were determined with a colorimeter (CR-400; Minolta, Kyoto, Japan). The Commission Internationale de l’Eclairage (CIE) parameters (L*, a*, b*) were determined on the same marked points on 10 fruits from each chamber at the beginning and after 6 months of CA storage. Color measurements were taken on the yellowest (not red) part of the fruit. L* represents the color brightness, a* represents the position between red (+) and green (-), and b* represents the scale between blue (-) and yellow (+).

The total color difference was calculated using the following equation:

Total color difference (ΔE) = ([Δa*]^2^ + [Δb*]^2^ + [ΔL*]^2^)^1/2^,

where ΔE is ‘very distinct’ for ΔE > 3, ‘distinct’ for 1.5 < ΔE < 3, and ‘non-distinct’ for ΔE < 1.5. 

### 2.4. Sample Preparation and Volatile Compound Extraction

VOC samples were collected after 2 and 6 months of storage and after 1 and 7 days of shelf life at 20 °C to simulate retail conditions. The apples were analyzed after only 2 months because the NA group of apples had a shorter shelf-life.

VOC sampling involved a dynamic flow system coupled with a purge and trap technique. Four replicate samples, each comprising one randomly chosen fruit, were placed in 0.5-L glass containers. A mass flow controller (SFC4200, Sensirion AG, Stäfa, Switzerland) introduced purified air into these containers at a steady rate of 30 mL/min for 30 min to achieve volatile equilibrium. The purge and trap system transferred VOCs from the gas phase to a conditioned sorbent tube (TDU, Gerstel, Mülheim an der Ruhr, Germany) packed with Tenax TA for subsequent gas chromatograph (GC) desorption. Tube sampling at a flow rate of 30 mL/min for 10 min resulted in 300 mL of total sample. The TDU tube was inserted into a MultiPurpose Sampler tray (MPS, Gerstel, Germany) for analysis.

### 2.5. Volatile Compound Identification and Quantification

VOCs were analyzed using a 7890A gas chromatograph and a 5975C mass spectrometer (Agilent Technologies, Santa Clara, CA, USA). The analytes were desorbed in low-split mode in a thermal desorption unit (TDU, Gerstel, Germany) with a helium flow rate of 50 mL min^−1^ at 250 °C for 10 min. A −100 °C PTV inlet (CIS 4, Gerstel, Germany) with a glass wool-filled liner cryogenically trapped the desorbed analytes. After desorption, the analytes were transferred to the GC column (DB-Wax, 60 m × 0.32 mm × 1 µm) in splitless mode by rapidly heating the CIS inlet to 275 °C with a 5 min hold time. Helium flowed at 1.5 mL min^−1^, serving as the analyte separation carrier gas. The column temperature was initially held at 40 °C for 5 min, then increased to 230 °C at 4 °C min^−1^ and held for 2 min. The mass selective detector operated in full-scan mode and covered a mass range from 30 to 250 mz^−1^. The ionization source was 70 eV, and both the source and the spectrometer interface were held at 230 °C.

For VOC identification, the de-convoluted mass spectrum was compared with the NIST mass spectral database and authentic standards when available. VOCs were quantified by calibration with known concentrations of authentic standard mixtures of ethyl acetate, ethanol, ethyl propionate, propyl propionate, hexanal, 2-methylbutyl acetate, ethyl hexanoate, 1-hexanol, butyl butanoate, butyl hexanoate, propyl acetate, hexyl 2-methylbutanoate, 2-methylpropyl acetate, pentyl acetate, hexyl hexanoate, ethyl 2-methylbutanoate, hexyl propionate, 2-methylbutyl 2-methylbutanoate, ethyl butanoate, 6-methyl-5-hepten-2-one, hexyl acetate, limonene, butyl 2-methylbutanoate, and benzaldehyde. Quantification was based on the butyl butanoate response factor if no standards were available. The response factor of limonene was used for α-farnesene. Table S1A provides a detailed list of the volatile organic compounds, including their retention times and the quantitative and qualitative ions used.

### 2.6. Calculation of the Odor Activity Values 

The odor activity value (OAV) is the ratio between individual compound concentrations and their odor threshold (OT) in the corresponding matrix (Table S2). Since most available OT compound data are determined in water, headspace VOC concentrations were utilized to estimate aqueous-phase concentrations using Henry’s coefficients. The applied Henry’s constants were averaged from four reported values, as per Sander [19].

### 2.7. Statistical Analysis

Statistical analyses were performed using SPSS software (version 23) and Excel 2022 software. Differences in maturity parameters and VOC production among the groups were evaluated using ANOVA, followed by Tukey’s post hoc test for multiple comparisons. Statistical significance was set at a *p*-value of less than 0.05, corresponding to a 95% confidence interval. Figures were generated using NCSS software (version 2024).

## 3. Results and Discussion

### 3.1. Ethylene Production and Respiratory Rate

After 2 months of storage and 1 day of shelf life, the NA ‘Gala’ apples exhibited significantly higher ethylene production compared to those stored under other conditions. However, this difference was not observed after 7 days of shelf life. No significant differences in ethylene production were noted between the investigated groups following 6 months of storage, regardless of measurement after 1 day or 7 days of shelf life (Table 1).

In contrast to the ethylene evolution rate, after 6 months of storage plus 7 days at 20 °C, apples stored under the CA-et condition had a significantly higher respiration rate than apples stored under CA conditions, indicating that ethanol treatments induced higher metabolic activity under CA conditions. Overall, the respiration rate decreased after 6 months of storage compared to the 2-month storage period (Table 1).

The results suggest that low pO_2_ suppresses ethylene production, which is consistent with previous studies [20]. The reduced ethylene production under low pO_2_ conditions can be attributed to decreased 1-aminocyclopropane-1-carboxylate (ACC)-oxidase activity, which is oxygen-dependent [21]. Moreover, the reduced oxygen concentrations in CA conditions slow down metabolism and delay ripening in apples [10,22,23], which aligns with our findings.

Our results indicated that ethanol and hexanal treatments did not significantly affect ethylene production after 2 months of storage, in contrast to several previous studies. Weber et al. [24] reported that ethanol application at a concentration of 0.3 mL ethanol/kg apples/month over 8 months of storage reduced ‘Royal Gala’ apples’ ethylene production after 7 days of shelf life. Similarly, Thewes et al. [25] found that ethanol vapor (500 ppm) inhibited ethylene production in ‘Nicoter’ and ‘Elstar’ apples. The discrepancy with our study may be due to varietal differences, specific storage conditions, or the ethanol concentrations used. Tiwari and Paliyath [26] demonstrated that when tomatoes are dipped in a 0.01% *v*/*v* hexanal solution, hexanal reduces the expression of ethylene biosynthesis genes.

Interestingly, the ethanol treatment led to a significantly higher respiration rate after 7 days of shelf life following 6 months of storage. This finding contrasts with that of Weber et al. [22], who observed that ethanol application during storage decreased both ethylene production and the respiration rate in ‘Braeburn’ apples, suggesting its effectiveness in delaying ripening processes. The higher respiration rate in our study may reflect a compensatory metabolic response, possibly due to ethanol treatment-induced stress. Furthermore, hexanal’s impact on the respiration rate is inconsistent with previous studies on other fruits. For example, Yuan et al. [27] reported that hexanal treatment reduced the respiration rate in strawberries.

### 3.2. Fruit Color

Color is an important quality parameter in fruit evaluation. There were no statistically significant differences in color parameters (L*, a*, b*) or total color difference (ΔE) between the ‘Gala’ apple groups after 6 months of storage. All groups showed a ΔE value higher than 3, indicating visible color changes. However, these changes did not differ significantly between the CA, CA-et, and CA-he groups (Table 2).

Previous studies have shown that treatments affect fruit color. For example, ethanol is known to prevent the degradation of green color in apples; in contrast, our results showed no significant change in the a* value, which represents the red–green color axis [10]. Sharma et al. found that pre-harvest spraying of sweet cherries with a hexanal solution kept the red color consistently high, while post-harvest exposure to hexanal did not significantly improve the color [28]. The sensory analysis in the study by Silué et al. showed that hexanal treatment improved the color of mango flesh [29]. In addition, Cheema et al. found that tomatoes dipped in hexanal solution showed higher L* values and hue angles, and lower red color intensity, as compared to control fruits during storage, suggesting delayed ripening [30]. Consistent with our results, Sriskantharajah et al. found that hexanal application as a pre-harvest spray did not cause significant differences in color parameters between the treated and control groups [9]. The absence of hexanal and ethanol effects on color in our study may be attributed to the relatively low concentration of the volatile aroma precursors added.

### 3.3. Volatile Compounds Analysis

The VOC profiles of the apples were evaluated after 2 and 6 months of cold storage in a CA, in a CA with ethanol added (CA-et), and in a CA with hexanal added (CA-he), and after 1 and 7 days at 20 °C. In addition, a VOC analysis of the apples stored in a normal air atmosphere (NA) at 1 °C for 2 months, plus 1 and 7 days at 20 °C, was performed. The VOC production (μg kg^−1^ h^−1^) results are summarized in Table 3.

After 2 months of storage plus 1 and 7 days of shelf life, 25 VOCs were identified in ‘Gala’ apples from the NA group, including 18 esters, 3 alcohols, and 4 other components. There were notable differences in the production and diversity of VOCs between the storage treatments.

#### 3.3.1. Total Volatile Compounds

**Figure 1:**
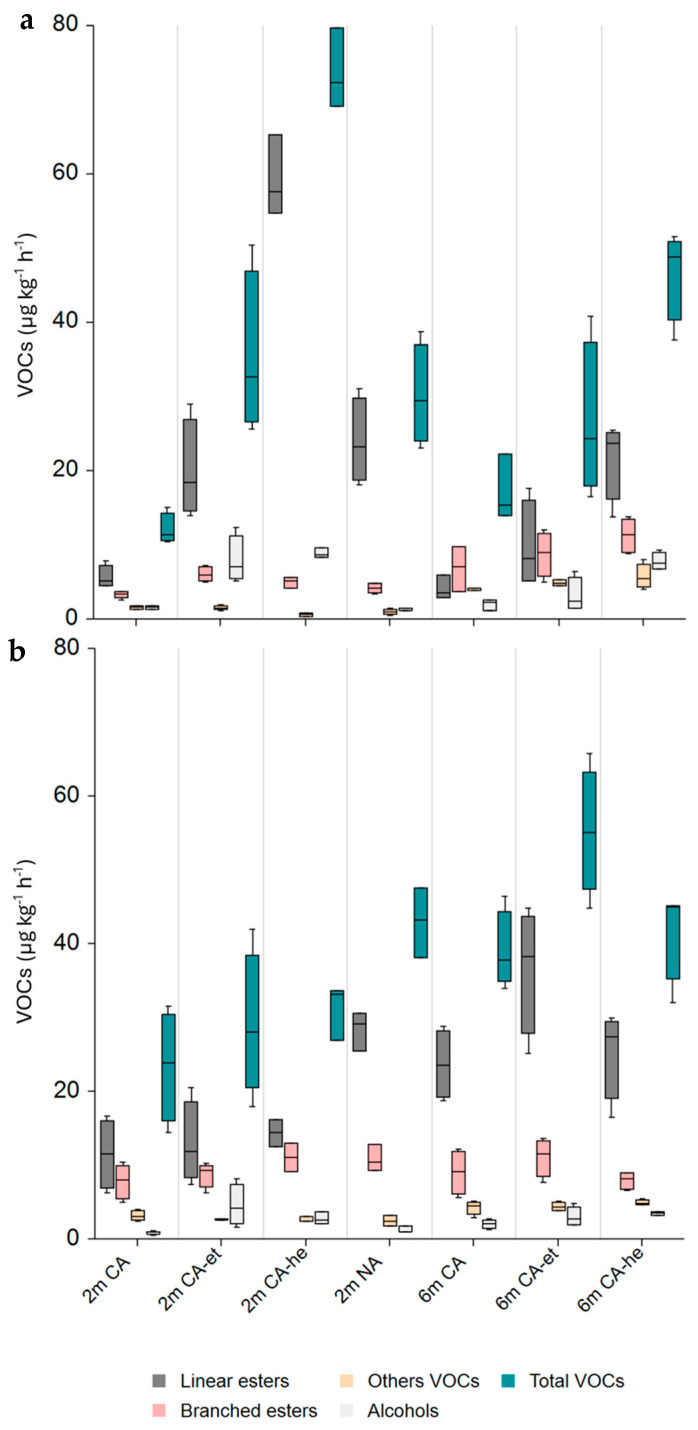
Volatile compound (VOC) production (μg kg^−1^ h^−1^) after 2 and 6 months of storage followed by 1 day (**a**) and 7 days (**b**) of shelf life. 2 m: 2 months of storage; 6 m: 6 months of storage; NA: normal air atmosphere; CA: controlled atmosphere; CA-et: controlled atmosphere with ethanol; CA-he: controlled atmosphere with hexanal.

After 2 months of storage plus 1 day of shelf life, the CA-he group of ‘Gala’ apples had significantly higher total VOC production compared to other treatments. This trend continued after 6 months of storage plus 1 day at 20 °C, and the CA-he group continued to produce more VOCs. In both cases, these differences were not detected after 7 days. The CA condition apples emitted the lowest VOC quantity after 2 months of storage and 1 day of shelf life, and there were no significant differences between the NA group and the CA-et group. Furthermore, after 6 months of storage followed by 7 days of shelf life, the CA-et group demonstrated significantly higher total VOC production compared to the CA group (Figure 1).

#### 3.3.2. Total Esters

Esters contribute the most to apple aroma, and butyl acetate, hexyl acetate, and 2-methyl butyl acetate are the most important esters in ‘Gala’ apples [31]. After 2 months of storage plus 1 day at 20 °C, the CA-he group of ‘Gala’ apples showed significantly higher total ester production compared to the other treatments, while the CA group of apples showed the lowest ester production. After 7 days at 20 °C, the NA group of apples produced the highest amount of total esters compared to the other groups. Similar patterns were observed after 6 months of storage, although the differences depended on the substrate added. After 1 day of shelf life, the CA-he group of apples had the highest ester production, but after 7 days of shelf life, the CA-et apple group had the highest total ester production.

Esters are synthesized by alcohol esterification with acyl-CoAs, a process catalyzed by AAT. Fatty acids degraded by beta-oxidation or lipoxygenase (LOX) serve as precursors for linear esters such as hexyl acetate, while amino acids lead to branched esters, such as 2-methylbutyl acetate and 2-methylpropyl acetate [32]. After 2 months of storage and 1 day of shelf life, the CA-he group of ‘Gala’ apples produced a significantly higher amount of linear esters. Similarly, the CA-et group of apples exhibited an elevated amount of linear esters compared to CA alone, indicating that hexanal or ethanol addition enhances linear ester production. However, no significant differences were observed between the CA-et and NA groups. After 2 months of storage plus 7 days at 20 °C, the NA group of apples exhibited the highest amount of linear esters.

After 2 and 6 months of storage, linear ester production showed differences after 1 and 7 days at 20 °C. The CA-he group showed the highest linear ester production, while the CA-et group showed a higher linear ester production compared to the CA apples after 6 months of storage and 7 days of shelf life. Branched ester production also showed differences. After 2 months of storage and 1 day of shelf life, the CA-et group of apples showed significantly higher branched ester production compared to the CA group, but it was not higher than other groups. These differences were no longer apparent after 7 days at 20 °C and were not observed after 6 months of storage.

#### 3.3.3. Individual Esters

Following 2 months of storage and 1 day of shelf life, the NA group demonstrated the highest linear ester production, including butyl acetate, butyl butanoate, hexyl propanoate, butyl hexanoate, hexyl butanoate, and hexyl hexanoate, in comparison to the other groups. The elevated production may be attributed to the increased ethylene production in the NA group of apples (Table 1). Given that ethylene production is influenced by pO_2_ levels, and ester formation is ethylene-dependent [32], this relationship likely accounts for the observed trend. However, after 7 days at 20 °C, this difference persisted for only a limited range of esters, with no significant differences observed for compounds such as butyl hexanoate, hexyl butanoate, and hexyl hexanoate. This suggests that certain enzymes involved in ester synthesis may regenerate following CA storage [33].

Apples produce ethyl acetate in predominantly elevated concentrations as a fermentation product under low-oxygen storage conditions. These environments restrict oxygen availability, thereby reducing ATP production and leading to anaerobic metabolite formation, including ethyl acetate [34]. While high ethyl acetate concentrations are generally undesirable due to their association with off flavors and adverse impacts on aroma, low concentrations can enhance apple aroma and flavor, with ethanol serving as a precursor for ethyl acetate synthesis [35]. After 2 months of storage and 1 day of shelf life, the CA-et group of apples had the highest ethyl acetate production, exceeding the NA group by more than 200 times. This indicates that ethanol in the ‘Gala’ apples was converted into ethyl acetate by the AAT enzyme. However, this increased production was no longer detectable after 7 days at 20 °C. Furthermore, no significant differences in ethyl acetate production were observed between the NA and CA groups after 2 months and 1 day of shelf life, indicating that pO_2_ levels did not induce anaerobic metabolism in the apples in CA storage. After 6 months of storage and 1 day of shelf life, the CA-et group again exhibited the highest ethyl acetate production, which notably remained elevated even after 7 days at 20 °C.

Pentyl acetate is another ester that showed changes due to ethanol or hexanal during storage in a CA. After 2 months of storage followed by 1 day at 20 °C, the CA-he group of apples produced more pentyl acetate compared to the CA group. After 6 months of storage with 1 day of shelf life, only the CA-he apples maintained elevated levels of pentyl acetate. However, these differences did not persist after 7 days of shelf life, regardless of whether the apples were stored for 2 or 6 months. Rowan et al. explained the biosynthetic pathway of pentyl esters from hexanal using deuterium-labeled fatty acids, C-6 aldehydes, and alcohols in ‘Granny Smith’ and ‘Red Delicious’ apples [16]. Their study showed that hexanal oxidation in apples leads to hexanoic acid formation, which can subsequently undergo α-oxidation to produce pentanoic acid. This pentanoic acid is then used to synthesize pentyl esters and pentanoate esters. Pentyl acetate has an odor threshold of about 43 µgL^−1^ and is associated with fruity and banana-like notes [36]; therefore, it is often used as a flavoring agent in the food and beverage industry, particularly for imparting a fruity, banana-like flavor on products such as candies, chewing gum, and soft drinks. In addition, pentyl acetate is used as a flavoring agent in perfume and fragrance production, contributing to pleasant and refreshing notes [37].

Ethyl hexanoate was detected in apples from the CA-et group only after 2 months of storage and 1 day of shelf life. After 6 months of storage, it was present only in trace amounts across all groups, with no significant differences observed between groups, regardless of shelf life duration.

Hexyl acetate was the most abundant ester in this study. Hexanal’s addition to the CA significantly increased hexyl acetate production in the ‘Gala’ apples, and the production was about fivefold higher compared to the NA group after 2 months of storage plus 1 day at 20 °C. Levels remained elevated after 6 months of storage but did not persist after 7 days at 20 °C, when the highest concentration of hexyl acetate was observed in the NA group. While the hexyl acetate concentration decreased after 6 months in all groups, it remained the highest in the CA-he group of apples, which showed a more than 10-fold higher hexyl acetate concentration than CA apples and about a 7-fold higher concentration than the CA-et group of apples. However, after 7 days at 20 °C, the differences between the CA, CA-et, and CA-he groups were no longer present. This indicates that the effect of hexanal decreased with time and that the AAT enzyme responsible for the formation of hexyl acetate may have regenerated after 7 days of storage under CA conditions. Hexyl acetate is associated with sweet, fruity, and floral descriptors and contributes significantly to the aroma of various apple varieties due to its relatively low odor threshold (2 µgL^−1^) [32,36]. The ‘Gala’ apple variety actively converted hexanal to hexyl acetate, as evidenced by the increased hexanol and hexyl ester production, a phenomenon also observed in ‘Golden Delicious’ apples [17].

Among the linear esters, the significant influence of hexanal or ethanol was observed also for butyl butanoate, hexyl hexanoate, and butyl hexanoate. The CA-he condition increased hexyl hexanoate threefold after 6 months of storage plus 1 day of shelf life compared to the CA. In contrast, apples from the CA-et group emitted the highest amount of butyl butanoate and butyl hexanoate as compared to the other groups after 6 months of storage plus 7 days of shelf life.

Ethyl 2-methylbutanoate plays a crucial role in the aroma and flavor of ‘Gala’ apples, contributing to consumer acceptance and the characteristic apple aroma [38]. The addition of ethanol to CA resulted in ethyl 2-methylbutanoate appearing after 2 months of storage and 1 day of shelf life. Ethyl 2-methylbutanoate was subsequently quantified only in the NA group after 2 months of storage plus 7 days at 20 °C.

2-Methylbutyl acetate is one of the principal esters in ‘Gala’ apples, as confirmed in this study [6]. Moreover, ethanol addition to the CA resulted in higher 2-methylbutyl acetate production after 2 months of storage plus 1 day at 20 °C, while hexanal had an effect on the increase in 2-methylbutyl acetate after 7 days at 20 °C compared to the CA. There were no differences among the groups after 6 months of storage.

2-Methylbutyl butanoate was formed within detectable limits in ‘Gala’ apples only after 6 months of storage, and both ethanol and hexanal showed effects. Notably, hexanal inhibited the synthesis of this compound after 6 months of storage and 1 day of shelf life at 20 °C, although this group of apples exhibited higher levels of 2-methyl-1-butanol, the precursor of 2-methylbutyl butanoate, compared to the CA group during this period. Conversely, ethanol addition resulted in higher 2-methylbutyl butanoate production after 6 months of storage plus 7 days at 20 °C.

Remarkably, the CA-he group of ‘Gala’ apples showed the highest hexyl 2-methylbutanoate production after 6 months of storage plus 1 day at 20 °C, with significantly higher values than those observed after 2 months of storage plus 1 day at 20 °C. This contrasts with the results of Matich and Rowan [39], who reported that hexyl 2-methylbutanoate production decreased with prolonged storage and was not significantly increased by incubation with d3-hexanol, although low concentrations of d3-hexyl 2-methylbutanoate were detected in ‘Red Delicious’ apples.

#### 3.3.4. Total Alcohols

Alcohols play a crucial role in apple aroma as esters precursors [4]. After 2 months of storage followed by 1 day of shelf life, the CA-et and CA-he treatment groups exhibited significantly higher production of total alcohols compared to the CA and NA groups (Figure 1). However, these differences were not observed with hexanal after 7 days at 20 °C, suggesting that the enzyme ADH rapidly converted hexanal into alcohol, and that both ethanol and hexanal were subsequently further metabolized. After 6 months of storage followed by 1 day of shelf life, only the CA-he treatment resulted in increased total alcohol production. However, no differences in total alcohols were detected between treatments after 7 days at 20 °C.

#### 3.3.5. Individual Alcohols

The CA-et ‘Gala’ apples only emitted higher ethanol production after 2 months of storage followed by 1 day at 20 °C and after 6 months of storage followed by 7 days at 20 °C, indicating that the ethanol was rapidly metabolized into other compounds in the apple tissue. Ethanol is metabolized in fruit tissue via two main pathways: conversion to acetaldehyde by ADH or esterification by the enzyme AAT [12]. Ethanol metabolism in apples is influenced by temperature and cultivar, as temperature affects ADH and AAT activity differently in different apple cultivars. AAT reportedly has different affinities for alcohol substrates depending on the apple variety [12,40]. The higher ethanol production after 6 months of storage plus 7 days at 20 °C also positively correlated with higher ethyl acetate, butyl hexanoate, butyl acetate, and 2-methyl butanoate production in these apples. The higher butyl acetate concentration could be explained by ADH’s ability to convert ethanol into acetaldehyde. Acetaldehyde is then further converted into acetic acid by the enzyme acetaldehyde dehydrogenase (ALDH). This reaction is also an oxidation process that usually requires oxygen for efficient completion. In the absence of oxygen, acetaldehyde can accumulate, which can lead to toxic effects. The enzyme acetic acid-CoA ligase (ACSL) catalyzes the conversion of acetic acid to acetyl-CoA [4,41]. Acetyl-CoA then serves as an important substrate for ester synthesis, which contributes significantly to fruit aroma.

Hexanal’s addition to the CA storage increased 2-pentanol formation in the ‘Gala’ apples after 2 months of storage followed by 1 day at 20 °C. After 6 months of storage, higher 2-pentanol production was observed after 7 days. The elevated 1-butanol concentration in the CA-et and CA-he groups after 2 months of storage plus 1 day of shelf life indicates that these precursors influence the metabolism of ‘Gala’ apples. After 2 months of storage, 1-butanol was no longer detected in either the NA or CA group after 1 and 7 days at 20 °C, presumably due to its conversion into butyl esters such as butyl propionate. This suggests that AAT played a role, converting 1-butanol into its corresponding ester in these apple groups.

2-Methyl-1-butanol is an important alcohol released by ‘Gala’ apples [34]. Both ethanol and hexanal were found to stimulate 2-methyl-1-butanol formation in the ‘Gala’ apples after 2 months of storage plus 1 day at 20 °C. Hexanal’s effect was also observed after 6 months of storage followed by 1 day at 20 °C. However, no significant differences were observed after 7 days of shelf life for both periods.

In the NA group, 1-hexanol was the most abundant alcohol after 2 months of storage and 1 day of shelf. As expected, hexanal’s addition to the CA significantly affected the production of 1-hexanol in the ‘Gala’ apples. Compared to the NA group, the CA-he group of apples showed about five times higher 1-hexanol production after 2 months of storage plus 1 day of shelf life. After 7 days of shelf life, the levels returned to their original values. This effect was even more pronounced after 6 months of storage, and it was also noticeable after 7 days. This indicates that hexanal was rapidly converted into the corresponding alcohol in the apple tissue under the influence of ADH. 1-Hexanol serves as a substrate for AAT and is involved in hexyl ester formation. The higher 1-hexanol concentration in the CA-he group probably contributed to the characteristically higher production of hexyl acetate, hexyl butanoate, and hexyl hexanoate esters in this group.

#### 3.3.6. Other Compounds

Regarding the other VOCs, the CA-he group notably showed a significantly lower concentration of total other compounds compared to the other groups (Figure 1). This was particularly evident after 2 months of storage plus 1 day at 20 °C, mainly due to the lower concentration of α-farnesene.

Hexanal can undergo two different metabolic pathways in apple tissue: it can be reduced to hexanol, which is then transformed into hexyl esters, or it can be oxidized to hexanoic acid, a precursor for hexanoate esters. Additionally, α-oxidation of hexanoic acid results in the formation of pentyl and pentanoate esters, whereas β-oxidation produces butanoic acid along with butyl and butanoate esters [16]. Furthermore, pentanoate esters may undergo hydrolysis to form alcohols, which can then oxidize to yield ketones, such as 2-pentanone, or reduce to form 2-pentanol. Two aldehydes, hexanal and benzaldehyde, were detected in the ‘Gala’ apples. The CA-he group of apples showed higher hexanal production only after 1 day at 20 °C following 2 and 6 months of storage. Under the influence of ADH, the aldehyde was rapidly converted to alcohol and then to ester by AAT. 2-Pentanone was exclusively present in the CA-he group, suggesting that hexanal influences the metabolism of these VOCs.

### 3.4. Odor Activity Values

More than 350 volatile aroma compounds have been identified in apples, but only 20-30 of these compounds contribute significantly to the characteristic aroma of apples [4]. The influence of a particular VOC on the overall aroma depends not only on its concentration but also on its OT. To evaluate the contribution of VOCs to the aroma of ‘Gala’ apple fruits, each VOC’s concentration was divided by the corresponding OT value reported in the literature, giving the odor activity value (OAV) [36]. A compound with an OAV greater than 1 is considered an important aroma compound. The higher the OAV is, the greater the VOC’s contribution to the overall aroma profile is. Table S2 gives the OT values in water for the VOCs in the ‘Gala’ apples. The OT values for 2-pentanol, propyl hexanoate, butyl octanoate, and α-farnesene were unknown and therefore not considered.

The main aroma compounds in ‘Gala’ apples are esters and aldehydes, and butyl acetate, hexyl acetate, and 2-methylbutyl acetate are crucial for the fruity and floral notes [42,43]. Although alcohols have not been identified as major contributors to apple aroma through GC-olfactometry methods [44], their concentrations are important to consider, as they are metabolic precursors to esters, which are key contributors to apple aroma. According to Young et al. [45], a mixture of 1-butanol with 2-methylbutyl acetate and hexyl acetate was found to closely replicate the red apple aroma characteristic of ‘Gala’ apples [45]. This indicates that alcohols might enhance the overall aroma in combination with other compounds rather than directly through their own odor activity.

**Figure 2:**
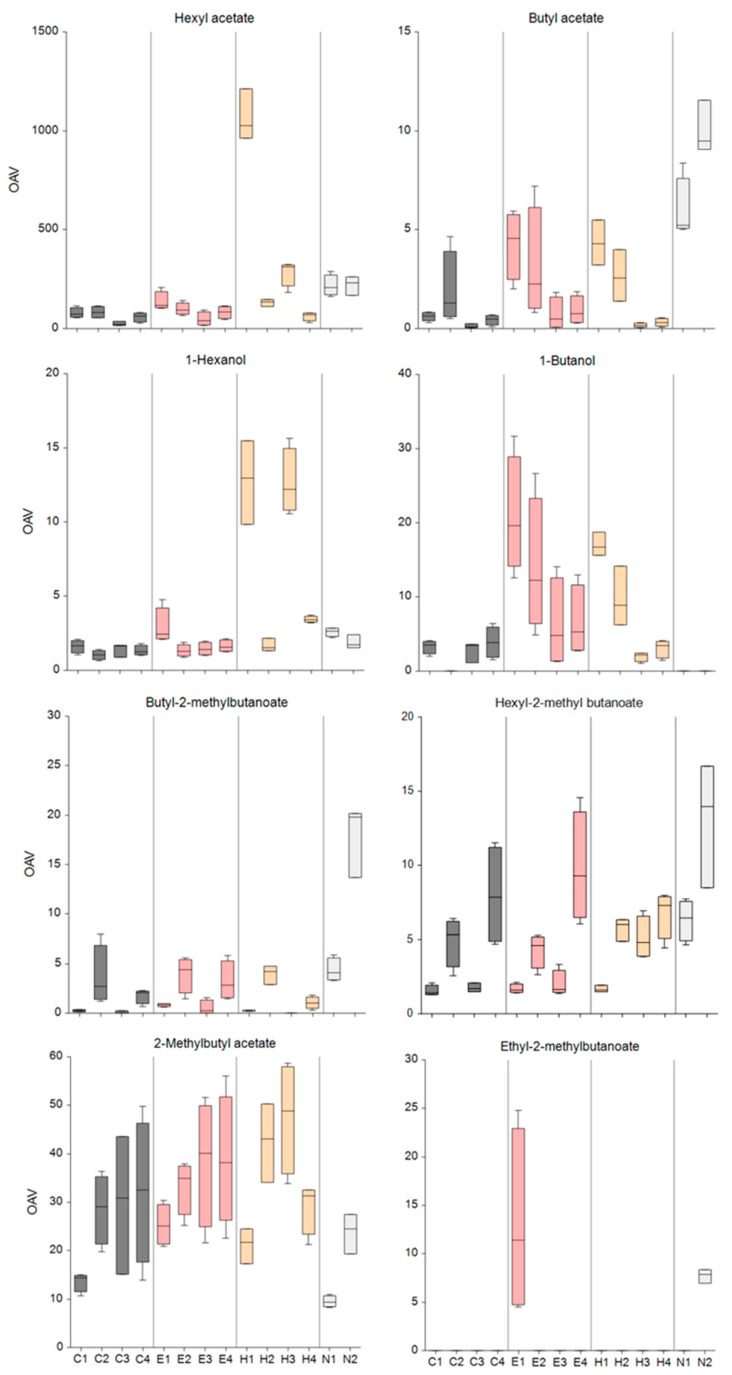
OAVs of the most important VOCs in ‘Gala’ apples after 2 and 6 months of storage followed by 1 day and 7 days of shelf life. C: controlled atmosphere; E: controlled atmosphere with ethanol; H: controlled atmosphere with hexanal; N: normal air atmosphere. Storage time: 1 (2 months of storage + 1 day of shelf life); 2 (2 months of storage + 7 days of shelf life); 3 (6 months of storage + 1 day of shelf life); 4 (6 months of storage + 7 days of shelf life).

In total, 9 of the 25 identified VOCs in this study exhibited an OAV exceeding 1 and had the most significant impact on the aroma profile in this investigation. These VOCs were hexyl acetate, 2-methylbutyl acetate, and 1-butanol, followed by butyl 2-methylbutylbutanote, hexyl 2-methylbutanoate, butyl acetate, ethyl 2-methylbutanoate, and 1-hexanol (Figure 2). Storage conditions and treatments exerted an influence on the volatile profile of the ‘Gala’ apples.

In this study, hexyl acetate, associated with sweet, fruity, and floral descriptors [4,36], was the primary contributor to the ‘Gala’ apples’ aroma. The highest hexyl acetate OAV was observed in the CA-he group, after 2 and 6 months of storage and 1 day of shelf life. After 7 days, hexanal’s effect on hexyl acetate’s OAV was no longer statistically significant. In the same period, hexanal also had a significantly increased effect on the 1-hexanol OAV. Additionally, hexanal significantly affected the 2-methyl-1-butanol OAV after 6 months of storage and 1 day of shelf life. The CA-he apples exhibited an increased total OAV compared to the other groups, primarily due to hexyl acetate.

The ethyl 2-methylbutanoate compound is characterized by a low OT (0.06 µgL^−1^ [31]) and confers a fruity aroma to apples [36]. It was previously identified as the most preferred compound in ‘Gala’ apples by consumers [38]. Ethanol’s addition to the CA resulted in ethyl 2-methylbutanoate production in the ‘Gala’ apples to such an extent that it significantly contributed to the ‘Gala’ apple aroma after 2 months of storage and 1 day of shelf life. This ester only contributed to the aroma in the NA group after 2 months of storage and 7 days of shelf life. Similarly, ethanol significantly affected the increased OAV of 2-methylbutyl acetate, one of the most important aroma compounds of ‘Gala’ apples, after 2 months of storage and 1 day of shelf life. Over the same period, this group of apples also had a greater aroma contribution from 1-butanol compared to the CA group.

Butyl acetate, characterized by fruity apple descriptors and an OT of 66 µgL^−1^, constitutes an important component of ‘Gala’ apple aroma [31,36]. Both ethanol and hexanal exhibited significant effects on the increased butyl acetate OAV levels after 2 months of storage and 1 day of shelf life compared to the CA alone, but not compared to the NA.

### 3.5. Correlation Analysis for Hexyl Acetate

**Figure 3:**
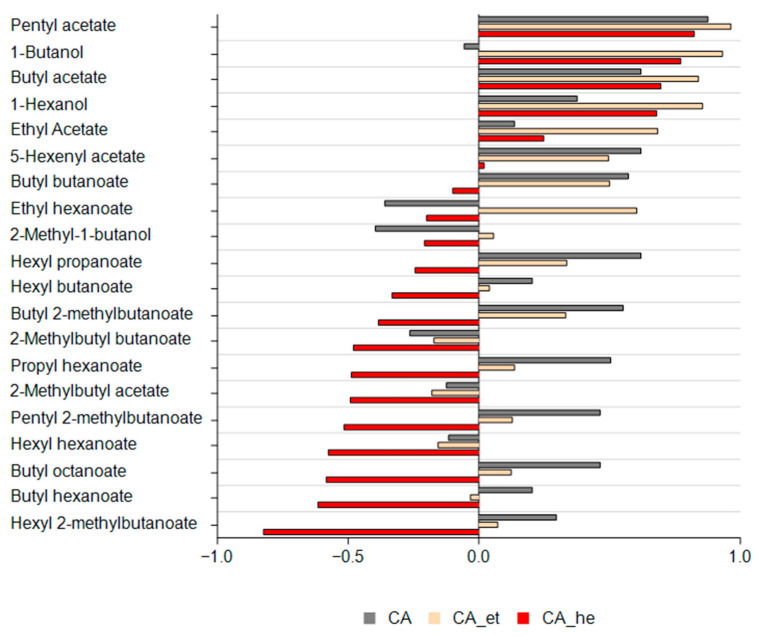
Pearson correlation coefficients between hexyl acetate and VOCs under different storage conditions. CA: controlled atmosphere; CA_et: controlled atmosphere with ethanol, CA_he: controlled atmosphere with hexanal.

Hexyl acetate was the primary contributor to the ‘Gala’ apple aroma in this study, so a correlation analysis of this compound with the remaining VOCs was conducted separately for each storage atmosphere. The Pearson coefficients for hexyl acetate are presented in Figure 3.

Hexanal exhibited a significant effect on hexyl acetate synthesis, as also reflected in the OAVs. Hexyl acetate demonstrated the strongest positive correlation with pentyl acetate, 1-butanol, and butyl acetate and the strongest negative correlation with hexyl 2-methylbutanoate, butyl hexanoate, and butyl octanoate in the CA-he atmosphere. In the CA-et group, hexyl acetate showed a positive correlation with pentyl acetate, 1-butanol, and 1-hexanol; in the CA group, hexyl acetate was positively correlated with pentyl acetate, hexyl propanoate, and butyl acetate.

Figure 3 illustrates a strong positive correlation between hexyl acetate and ethyl hexanoate in the CA-et storage atmosphere, suggesting that increased hexyl acetate is accompanied by a corresponding rise in ethyl hexanoate under these conditions. Ethyl hexanoate has been linked to enhanced apple juice sweetness and was identified as one of the key compounds contributing to odor-associated sweetness enhancement [46]. The addition of ethanol into the CA environment may promote the formation of ethyl hexanoate, potentially enhancing the perceived sweetness of apples and apple-derived products.

Figure 3 demonstrates that hexyl acetate exhibited negative correlations with 14 out of 20 VOCs in the CA-he group, whereas in the CA and CA-et groups, it displayed negative correlations with 5 and 4 VOCs, respectively. This suggests that the addition of ethanol and hexanal to the CA affected the enzymes and synthetic pathways of aromatic compound formation in ‘Gala’ apples. However, importantly, hexanal significantly contributed to the overall aroma by enhancing the synthesis of hexyl acetate, a key compound in apple aroma. This increased synthesis played a crucial role in shaping the final aroma profile, ultimately outweighing the negative associations with other volatile compounds, as the CA-he apples always had an increased or equal total OAV compared to the other conditions.

Previous research has demonstrated that CA storage, particularly at lower oxygen levels, reduces the emission of volatile esters and fruity aromas in ‘Gala’ apples [44,47]. Key esters, such as butyl acetate, hexyl acetate, and 2-methylbutyl acetate, which define the characteristic ‘Gala’ apple flavor, are significantly reduced under CA conditions [8,44]. This decrease is attributed to lower enzymatic activity of AAT and LOX [47]. Storage in a standard CA resulted in higher levels of key volatiles (butyl acetate, 2-methylbutyl acetate, and hexyl acetate) compared to fruit stored under dynamic controlled atmosphere (DCA) chlorophyll fluorescence (CF) conditions. However, fruits stored under a DCA with a respiratory quotient (RQ) of 1.5 and RQ 2.0 also showed increased amounts of key volatile compounds, with a notable rise in ethanol and ethyl acetate, though still below the odor threshold [30]. Additionally, Both et al. [40] demonstrated that apples stored at 0.7 kPa O_2_ had higher levels of 1-hexanol compared to those stored at 0.5 kPa O_2_ [8]. 1-Hexanol is a precursor to hexyl acetate, which explains the reduced production of hexyl acetate in fruit stored at lower oxygen levels.

Storage conditions and treatments can influence the volatile profile of ‘Gala’ apples. In this study, hexyl acetate had the highest OAV and contributed the most to the ‘Gala’ apple aroma, as it reached OAVs higher than 1 in all groups, closely followed by 2-methylbutyl acetate. Importantly, our findings show that hexanal addition significantly increased the hexyl acetate production in apples, whereas ethanol primarily influenced the content of 2-methylbutyl acetate and ethyl 2-metyhlbutanoate, as discussed in Section 3.3.

In general, hexanal and ethanol exhibited a more pronounced effect on aroma after 2 months of storage compared to after 6 months of storage. This suggests that the influence of these volatiles on aroma biosynthesis is time-dependent, potentially due to changes in metabolic activity over extended storage periods. It is well established that fruit VOCs are derived from precursor molecules such as fatty acids, amino acids, and carbohydrates, which are transformed into aroma-related metabolites through enzymatic pathways. Considering that a precursor was consistently available to the apples throughout the storage period, we can infer that the limiting factor for aroma synthesis is not the presence of substrates but rather the enzymatic activity responsible for the conversion of precursors into aromatic compounds. Enzymatic activity in VOC production is known to decline over time due to factors such as reduced enzyme expression and alterations in cellular metabolism associated with prolonged cold storage [4,47].

Moreover, in this study, we observed that after 7 days of shelf life, there were no significant differences in VOC production between the treated and control apple groups compared to 1 day of shelf life. This suggests that the hexanal and ethanol treatments had transient effects on aroma compound biosynthesis, which diminished once the apples were removed from the CA storage conditions and exposed to ambient air at 20 °C. While hexanal and ethanol initially modulated aroma-related pathways, the apples’ endogenous regulatory mechanisms, such as feedback inhibition or substrate depletion, may have rebalanced metabolic fluxes after extended exposure to shelf conditions. This aligns with previous studies which indicate that VOC fluctuations tend to stabilize after apples are removed from a precursor atmosphere [17,18]. Future studies should investigate the specific enzymatic and genetic responses of apples to these treatments over time to better understand the mechanisms governing VOC synthesis and retention under hexanal- and ethanol-enriched CA storage.

## 4. Conclusions

In this study, ethanol- and hexanal-enriched CA atmospheres had a significant effect on the formation of aromatic compounds in ‘Gala’ apples, without affecting ethylene production and color change. Even at low concentrations, the ethanol and hexanal storage treatments increased VOC contents, especially esters and alcohols, which in turn led to significant changes in the OAVs of the ‘Gala’ apple aroma. This approach presents a promising strategy for enhancing apple aroma and improving fruit sensory quality during short-term CA storage.

## Figures and Tables

**Table 1:** Ethylene production rate, respiratory rate (CO_2_), and flesh firmness of ‘Gala’ apples after 2 and 6 months of storage and after 1 and 7 days of shelf life.

**1 day of shelf life**					
**Months of storage**		**NA**	**CA**	**CA-et**	**CA-he**
		**avg** **±** **sd**	**avg** **±** **sd**	**avg** **±** **sd**	**avg** **±** **sd**
Ethylene (µL kg^−1^ h−^1^)	±2	218.03 ± 73.77b	15.12 ± 26.76a	49.55 ± 24.71a	1.51 ± 0.38a
	6		6.74 ± 1.27a	11.32 ± 6.12a	5.09 ± 1.90a
CO_2_ (μg kg^−1^s^−1^)	2	8.25 ± 0.25a	6.35 ± 0.36b	8.77 ± 1.11a	8.34 ± 0.60a
	6		3.95 ± 0.77a	3.96 ± 0.58a	3.45 ± 0.50a
**7 days of shelf life**					
		**NA**	**CA**	**CA-et**	**CA-he**
		**avg** **±** **sd**	**avg** **±** **sd**	**avg** **±** **sd**	**avg** **±** **sd**
Ethylene (µL kg^−1^ h−^1^)	2	199.97 ± 59.45a	133.80 ± 48.52a	173.94 ± 71.52a	108.96 ± 66.79a
	6		85.77 ± 14.44a	95.29 ± 28.28a	119.45 ± 45.40a
CO_2_ (μg kg^−1^s^−1^)	2	10.90 ± 1.31a	11.93 ± 1.99a	10.01 ± 1.16a	11.39 ± 2.23a
	6		7.79 ± 0.44a	10.31 ± 1.50b	8.01 ± 1.02a

avg: average; sd: standard deviation. Means followed by the same letters in the same storage months and shelf days do not differ by the Tukey test at 5% probability.

**Table 2:** CIE (L*, a*, b*) parameters and ΔE for ‘Gala’ apples after 6 months of storage.

	CA	CA-et	CA-he
	avg ± sd	avg ± sd	avg ± sd
ΔL*^2^	17.84 ± 24.98	54.90 ± 87.58	48.37 ± 73.66
Δa*^2^	27.75 ± 33.53	39.18 ± 47.27	25.17 ± 39.11
Δb*^2^	12.78 ± 6.96	10.95 ± 9.94	8.15 ± 7.17
ΔE	5.58 ± 4.26	8.60 ± 4.91	6.38 ± 6.12

avg: average; sd: standard deviation.

**Table 3:** VOC production (μg kg^−1^ h^−1^) by ‘Gala’ apples (*n* = 4).

		1 Day of Shelf Life				7 Days of Shelf Life			
		NA	CA	CA-et	CA-he	NA	CA	CA-et	CA-he
VOC	Month of Storage	avg ± sd	avg ± sd	avg ± sd	avg ± sd	avg ± sd	avg ± sd	avg ± sd	avg ± sd
**Linear esters**									
Ethyl acetate	2	0.33 ± 0.16a	0.18 ± 0.04a	79.40 ± 35.82b	1.35 ± 0.62a	2.09 ± 0.84a	1.19 ± 1.31a	3.01 ± 0.52a	0.82 ± 0.31a
	6		1.26 ± 0.58a	16.07 ± 13.01b	2.37 ± 1.07a		0.56 ± 0.25a	0.98 ± 0.57b	0.43 ± 0.17a
Butyl acetate	2	65.55 ± 24.52a	6.28 ± 1.98b	43.82 ± 18.88a	44.20 ± 12.19a	112.12 ± 25.98b	18.96 ± 16.45a	32.41 ± 30.39a	26.52 ± 12.58a
	6		1.25 ± 1.19a	7.42 ± 8.41a	1.64 ± 1.04a		4.71 ± 2.72a	9.42 ± 7.26a	3.09 ± 2.11a
Butyl propionate	2	6.23 ± 1.73b	0.00 ± 0.00a	0.00 ± 0.00a	0.00 ± 0.00a	9.31 ± 1.20b	0.00 ± 0.00a	0.00 ± 0.00a	0.00 ± 0.00a
	6		0.00 ± 0.00a	0.00 ± 0.00a	0.00 ± 0.00a		0.00 ± 0.00a	0.00 ± 0.00a	0.00 ± 0.00a
Pentyl acetate	2	5.60 ± 2.15ab	3.33 ± 0.40a	7.07 ± 2.43ab	9.16 ± 1.69b	8.99 ± 0.81a	4.72 ± 1.81b	5.34 ± 1.56b	5.28 ± 0.48b
	6		0.85 ± 0.46a	2.06 ± 1.47a	5.88 ± 1.36b		2.68 ± 1.17a	4.39 ± 2.22a	1.78 ± 1.09a
Butyl butanoate	2	19.27 ± 7.22b	1.12 ± 0.50a	4.24 ± 0.94a	3.19 ± 1.14a	18.80 ± 0.93b	7.42 ± 4.31a	7.62 ± 3.28a	6.47 ± 0.99a
	6		0.46 ± 0.45a	2.11 ± 2.28a	0.00 ± 0.00a		4.87 ± 2.03ab	8.40 ± 3.47a	3.18 ± 1.97b
Ethyl hexanoate	2	0.00 ± 00a	0.00 ± 0.00a	0.78 ± 0.53b	0.00 ± 0.00a	0.00 ± 0.00a	0.00 ± 0.00a	0.00 ± 0.00a	0.00 ± 0.00a
	6		0.20 ± 0.35a	0.00 ± 0.00b	0.00 ± 0.00a		0.00 ± 0.00a	0.18 ± 0.13a	0.06 ± 0.12a
Hexyl acetate	2	133.69 ± 46.37a	46.61 ± 12.91c	78.37 ± 30.68ac	608.72 ± 61.38b	135.24 ± 16.89b	47.57 ± 14.22a	56.44 ± 20.04a	74.56 ± 7.16a
	6		14.22 ± 6.73a	28.02 ± 20.17a	159.55 ± 32.92b		34.95 ± 13.55a	48.30 ± 16.49a	36.12 ± 14.47a
Propyl hexanoate	2	0.24 ± 0.10b	0.00 ± 0.00a	0.16 ± 0.06a	0.00 ± 0.00a	1.27 ± 0.33a	0.94 ± 0.40a	0.81 ± 0.30a	0.74 ± 0.12a
	6		0.00 ± 0.00a	0.00 ± 0.00a	0.00 ± 0.00a		0.77 ± 0.37a	1.30 ± 0.42a	0.49 ± 0.56a
Hexyl propanoate	2	4.26 ± 0.73b	0.24 ± 0.13a	0.46 ± 0.20a	0.99 ± 0.33a	4.26 ± 1.07b	1.70 ± 0.70a	1.83 ± 0.66a	1.64 ± 0.02a
	6		0.17 ± 0.07a	0.36 ± 0.40a	0.64 ± 0.42a		1.35 ± 0.51a	1.55 ± 0.46a	1.52 ± 0.73a
Butyl hexanoate	2	26.95 ± 12.67b	2.63 ± 0.97a	6.58 ± 1.39a	1.48 ± 0.78a	30.39 ± 6.04a	22.17 ± 5.32ab	18.49 ± 3.80b	21.71 ± 2.86ab
	6		3.83 ± 2.42a	10.21 ± 8.36a	1.04 ± 0.45a		42.92 ± 6.68a	76.26 ± 9.52b	28.53 ± 11.56a
Hexyl butanoate	2	16.17 ± 5.96a	2.80 ± 0.63a	4.25 ± 1.05a	7.65 ± 2.12a	13.81 ± 6.06a	7.81 ± 1.70ab	6.58 ± 1.58b	7.35 ± 1.46ab
	6		2.95 ± 0.76a	4.31 ± 2.30a	5.99 ± 2.02a		16.55 ± 4.44ab	23.15 ± 3.44a	15.26 ± 4.00b
Hexyl hexanoate	2	12.92 ± 7.04b	3.42 ± 1.32a	3.73 ± 1.38a	2.47 ± 1.54a	12.57 ± 7.73a	15.22 ± 5.46a	10.07 ± 0.36a	16.18 ± 2.95a
	6		25.19 ± 10.09a	43.85 ± 11.70ab	69.11 ± 15.23b		176.36 ± 45.79a	256.59 ± 41.36a	201.76 ± 2.06a
Butyl octanoate	2	3.29 ± 1.67b	0.45 ± 0.16a	0.99 ± 0.39a	0.17 ± 0.12a	4.63 ± 2.02a	2.92 ± 0.88a	2.68 ± 0.96a	2.98 ± 0.21a
	6		0.27 ± 0.27a	1.01± 0.76a	0.15 ± 0.07a		2.69 ± 0.55a	5.00 ± 1.32b	1.93 ± 0.86a
**Branched esters**									
2-Methylpropyl acetate	2	0.00 ± 0.00a	0.00 ± 0.00a	1.27 ± 0.47b	0.00 ± 0.00a	0.00 ± 0.00b	3.04 ± 1.14a	2.99 ± 0.55a	3.76 ± 0.40a
	6		0.00 ± 0.00a	0.00 ± 0.00a	0.00 ± 0.00a		0.00 ± 0.00a	0.00 ± 0.00a	0.00 ± 0.00a
Ethyl 2-methylbutanoate	2	0.00 ± 0.00a	0.00 ± 0.00a	0.22 ± 0.17b	0.00 ± 0.00a	0.14 ± 0.02b	0.00 ± 0.00a	0.00 ± 0.00a	0.00 ± 0.00a
	6		0.00 ± 0.00a	0.00 ± 0.00a	0.00 ± 0.00a		0.00 ± 0.00a	0.00 ± 0.00a	0.00 ± 0.00a
2-Methylbutyl acetate	2	24.99 ± 5.57a	34.80 ± 4.43ac	62.19 ± 12.89b	52.10 ± 10.44bc	63.91 ± 14.58a	71.94 ± 10.28a	81.33 ± 14.56ab	103.47 ± 13.68b
	6		79.35 ± 40.76a	99.01 ± 34.09a	117.53 ± 34.65a		85.57 ± 42.23a	101.42 ± 42.39a	71.33 ± 14.97a
Butyl 2-methylbutanoate	2	8.63 ± 3.11a	0.47 ± 0.20a	1.53 ± 0.34b	0.49 ± 0.17b	35.61 ± 4.92a	6.58 ± 4.75a	7.30 ± 3.58a	7.21 ± 1.38a
	6		0.16 ± 0.28a	0.97 ± 1.33a	0.00 ± 0.00a		3.45 ± 1.52a	6.13 ± 3.52a	1.96 ± 1.18a
2-Methylbutyl butanoate	2	0.00 ± 0.00a	0.00 ± 0.00a	0.00 ± 0.00a	0.00 ± 0.00a	0.00 ± 0.00a	0.00 ± 0.00a	0.00 ± 0.00a	0.00 ± 0.00a
	6		0.47 ± 0.09a	0.38 ± 0.26a	0.00 ± 0.00b		1.48 ± 0.10a	1.98 ± 0.21b	1.50 ± 0.16a
Amyl 2-methylbutanoate	2	0.26 ± 0.05b	0.00 ± 0.00a	0.00 ± 0.00a	0.00 ± 0.00a	1.12 ± 0.33b	0.41 ± 0.18a	0.40 ± 0.13a	0.37 ± 0.04a
	6		0.00 ± 0.00a	0.00 ± 0.00a	0.00 ± 0.00a		0.34 ± 0.12a	0.47± 0.19a	0.16 ± 0.11a
5-Hexenyl acetate	2	0.69 ± 0.26a	0.38 ± 0.05a	0.69 ± 0.20a	0.74 ± 0.09a	0.85 ± 0.10a	0.76 ± 0.13a	0.79 ± 0.17a	1.05 ± 0.13a
	6		0.22 ± 0.01a	0.37 ± 0.18a	0.87 ± 0.11b		0.67 ± 0.26a	1.05 ± 0.29a	0.62 ± 0.29a
Hexyl 2-methylbutanoate	2	16.10 ± 4.08b	3.84 ± 0.89a	3.99 ± 0.87a	3.96 ± 0.46a	33.52 ± 9.31b	12.04 ± 3.36a	10.23 ± 2.96a	13.74 ± 2.07a
	6		4.48 ± 1.02a	4.95 ± 1.89a	12.18 ± 3.60a		20.25 ± 8.73a	24.03 ± 7.35a	16.35 ± 4.82a
**Alcohols**									
Ethanol	2	2.67 ± 1.21a	3.52 ± 0.83a	35.14 ± 16.47b	4.93 ± 2.57a	4.14 ± 3.50a	1.68 ± 0.42a	3.35 ± 1.84a	2.52 ± 0.65a
	6		5.21 ± 1.48a	5.42 ± 1.13a	6.38 ± 0.77a		6.61 ± 1.03a	10.13 ± 1.33b	5.99 ± 0.26a
2-Pentanol	2	0.00 ± 0.00a	0.00 ± 0.00a	0.00 ± 0.00a	8.90 ± 7.88b	0.00 ± 0.00a	0.00 ± 0.00a	12.10 ± 14.07a	0.32 ± 0.04a
	6		0.00 ± 0.00a	6.82 ± 12.42a	13.80 ± 2.08a		0.00 ± 0.00a	0.00 ± 0.00a	8.81 ± 1.33b
1-Butanol	2	0.00 ± 0.00a	6.53 ± 1.44a	40.71 ± 17.06b	33.05 ± 5.24a	0.00 ± 0.00a	0.00 ± 0.00a	27.2 ± 18.78b	18.70 ± 7.31b
	6		5.49 ± 2.72a	12.39 ± 11.44a	3.68 ± 1.14a		7.95 ± 4.36a	13.05 ± 9.02a	6.03 ± 2.52a
2-Methyl-1-butanol	2	0.91 ± 0.12a	1.87 ± 0.28ab	3.69 ± 1.42c	3.32 ± 0.39bc	2.89 ± 0.28a	2.88 ± 0.88a	3.81 ± 0.81a	3.20 ± 0.34a
	6		6.84 ± 1.90a	6.61 ± 1.78a	12.57 ± 3.51b		4.27 ± 1.71a	5.86 ± 3.11a	5.00 ± 1.24a
1-Hexanol	2	11.25 ± 2.08a	6.75 ± 1.61a	12.01 ± 5.51a	52.01 ± 10.98b	8.21 ± 1.30a	4.28 ± 0.97b	5.39 ± 1.81ab	6.78 ± 1.67ab
	6		6.04 ± 1.75a	6.11 ± 1.87a	51.08 ± 4.80b		5.69 ± 1.14a	6.89 ± 1.65a	14.02 ± 1.76a
**Other VOCs**									
2-Pentanone	2	0.00 ± 0.00a	0.00 ± 0.00a	0.14 ± 0.04b	1.11 ± 0.40b	0.00 ± 0.00a	0.00 ± 0.00a	0.00 ± 0.00a	0.38 ± 0.05b
	6		0.00 ± 0.00a	0.00 ± 0.00a	12.11 ± 5.09b		0.00 ± 0.00a	0.00 ± 0.00a	2.44 ± 1.14b
Hexanal	2	0.00 ± 0.00a	0.00 ± 0.00a	0.00 ± 0.00a	0.57 ± 0.09b	0.00 ± 0.00a	0.00 ± 0.00a	0.00 ± 0.00a	0.00 ± 0.00a
	6		1.99 ± 0.99a	0.98 ± 0.39a	3.13 ± 1.06b		0.78 ± 0.29a	0.78 ± 0.21a	0.66 ± 0.14a
6-Methyl-5-heptene-2-one	2	0.15 ± 0.06a	0.12 ± 0.02a	0.16 ± 0.05a	0.09 ± 0.08a	0.17 ± 0.06a	0.40 ± 0.19a	0.35 ± 0.05a	0.44 ± 0.10a
	6		1.90 ± 0.47a	2.46 ± 0.69a	1.81 ± 0.35a		1.04 ± 0.45a	1.74 ± 0.16b	2.22 ± 0.31b
Benzaldehyde	2	0.27 ± 0.10a	0.26 ± 0.03a	0.26 ± 0.08a	0.21 ± 0.15a	0.20 ± 0.06a	0.21 ± 0.06a	0.33 ± 0.28a	0.32 ± 0.31a
	6		0.68 ± 0.07a	0.70 ± 0.11a	0.80 ± 0.09a		0.46 ± 0.04a	1.58 ± 2.06a	0.53 ± 0.10a
Estragole	2	2.61 ± 1.22a	4.62 ± 1.52a	5.23 ± 1.76a	2.17 ± 0.42a	7.38 ± 3.90a	9.20 ± 2.90a	8.72 ± 2.38a	7.00 ± 0.92a
	6		11.77 ± 3.30a	9.53 ± 1.89a	10.50 ± 3.78a		7.28 ± 3.97a	7.52 ± 4.17a	3.75 ± 1.40a
α-Farnesene	2	9.29 ± 6.16ab	13.93 ± 3.37a	11.18 ± 2.39ab	2.95 ± 2.85b	23.28 ± 7.46a	28.26 ± 7.87a	20.53 ± 1.52a	23.90 ± 5.07a
	6		35.89 ± 7.59a	45.56 ± 5.45a	40.12 ± 8.78a		43.94 ± 16.46a	42.61 ± 8.02a	47.65 ± 3.57a

avg: average; sd: standard deviation. Means followed by the same letters in the same month of storage and day of shelf life do not differ by the Tukey test at 5% probability.

## Data Availability

The original contributions presented in this study are included in the article/Supplementary Materials. Further inquiries can be directed to the corresponding author.

## Supplementary Materials

The following supporting information can be downloaded at: https://www.mdpi.com/article/10.3390/foods14060930/s1, Table S1: Identification, retention time, quantitative and qualitative ions for volatile compounds used in the study; Table S2: Mean heaspace volatile compounds concentration (*n* = 4) of ‘Gala’ apples. References [48,49] are cited in Supplementary Materials.

## Abbreviations

The following abbreviations are used in this manuscript:

VOC	Volatile compound
AAT	Alcohol acyltransferase
CA	Controlled atmosphere
ADH	Alcohol dehydrogenase
CA-et	CA-ethanol
CA-he	CA-hexanal
NA	Normal air atmosphere
CIE	Commission Internationale de l’Eclairage
OT	Odor threshold
AAC	1-Aminocyclopropane-1-carboxylate
ACSL	Acetic acid-CoA ligase
ALDH	Acetaldehyde dehydrogenase
OAV	Odor activity value
LOX	Lipoxygenase
DCA	Dynamic controlled atmosphere
CF	Chlorophyll fluorescence
RQ	Respiratory quotient
