# Supplementary material for: Comparative Analysis of Aroma Emissions in ‘Gala’ Apples Stored in Ethanol- and Hexanal-Enriched Controlled Atmosphere
Source: Foods. 2025 Mar 9;14(6):930. doi: 10.3390/foods14060930 (PMC11941499; doi:10.3390/foods14060930)
Supplement: Supplementary file 1 [file foods-14-00930-s001.zip › foods-3501822-supplementary.pdf]

Supplementary material

Table S1: Identification, retention time, quantitative and qualitative ions for volatile compounds used in the study.

**Identification**	Retention Time (min)	Quantitative (first) and qualitative ions (*m/z*)
Ethyl Acetate	10.31	61.0. 70.1, 87.9
Ethanol	11.96	31.1, 45.1
2-Pentanone	13.76	86.2, 71.1, 58.1
2-Methylpropyl acetate	15.05	73.1, 61.1, 86.2, 101.2
Ethyl 2-methylbutanoate	16.62	102.1, 57.1, 85.1, 115.0
Butyl acetate	17.52	61.0, 73.0, 87.0
Hexanal	18.00	72.1, 82.1, 67.1
2-Pentanol	19.17	73.2, 58.1, 87.2
2-Methylbutyl acetate	19.49	70.1, 55.1, 85.1, 101.0
1-Butanol	20.19	56.1, 41.1, 73
Butyl propoionate	20.21	75.0, 87.1, 101.1
Pentyl acetate	21.52	70.2, 61.1, 101.1
2-Methyl-1-butanol	22.61	70.1, 56.1, 42.1
Butyl butanoate	23.22	71.1, 89.1, 101.0, 116.1
Butyl 2-methylbutanoate	23.72	103.1, 85.1, 74.1, 130.1
Ethyl hexanoate	23.82	88.0, 99.1, 60.0, 73.0
2-Methylbutyl butanoate	24.45	71.1, 55.0, 89.0
Hexyl acetate	25.02	84.0, 61.0, 69.0
Propyl hexanoate	26.96	99.0, 117.1, 71.1, 87.0
Pentyl 2-methylbutanoate	27.27	103.1, 85.0, 70.1
5-Hexenyl acetate	27.34	67.0, 82.0, 54.1
Hexyl propanoate	27.69	75.1, 84.1, 69.1, 129.1
6-Methyl-5-heptene-2-one	27.91	108.2, 69.1, 129.1
1-Hexanol	28.03	56.1, 69.1, 84.1
Butyl hexanoate	30.30	99.1, 117.1, 71.1
Hexyl butanoate	30.38	71.1, 89.1, 84.1
Hexyl 2-methylbutanoate	30.72	103.1, 85.1, 69.1
Benzaldehyde	34.98	105.0, 77.0, 55.1, 73.0
Hexyl hexanoate	36.68	117.1, 99.1, 84.1
Butyl octanoate	36.79	127.1, 145.1, 73.0, 200.1
Estragole	39.20	148.1, 121.1 117.1
α-Farnesene	40.86	93.1, 107.1, 119.1, 189.2

Table S2: Mean heaspace volatile compounds concentration (n=4) of 'Gala' apples.

		1 day of shelf life at 20°C								7 days of shelf life at 20°C							
		NA		CA		CA-et		CA-he		NA		CA		CA-et		CA-he	
VOC	Month of storage	avg	sd	avg	sd	avg	sd	avg	sd	avg	sd	avg	sd	avg	sd	avg	sd
**Linear esters**																	
Ethyl Acetate (13500[a])*	2	0.03a	0.01	0.02a	0.00	6.87b	2.89	0.12a	0.05	0.16a	0.05	0.11a	0.13	0.52a	0.87	0.07a	0.03
	6			0.10a	0.05	1.38b	1.16	0.21a	0.09			0.05a	0.02	0.08b	0.05	0.04a	0.01
Butyl acetate (66[a])	2	5.28a	1.42	0.53b	0.20	3.78a	1.52	3.83a	1.01	8.88b	1.18	1.71a	1.66	2.77a	2.51	2.34a	1.16
	6			0.10a	0.09	0.63a	0.75	0.14a	0.09			0.39a	0.22	0.80a	0.65	0.26a	0.18
Butyl propionate (25[g])	2	0.51b	0.11	0.00a	0.00	0.00a	0.00	0.00a	0.00	0.74b	0.07	0.20a	0.17	0.00a	0.00	0.00a	0.00
	6			0.00a	0.00	0.00a	0.00	0.00a	0.00			0.00a	0.00	0.00a	0.00	0.00a	0.00
Pentyl acetate (43[b])	2	0.45ac	0.13	0.28a	0.05	0.61bc	0.19	0.79b	0.10	0.72a	0.04	0.41a	0.20	0.46a	0.12	0.46a	0.06
	6			0.07a	0.04	0.17a	0.13	0.52b	0.13			0.22a	0.10	0.37a	0.20	0.15a	0.09
Butyl butanoate (100l[a])	2	1.57b	0.55	0.09a	0.04	0.37a	0.07	0.28a	0.10	1.51b	0.16	0.65a	0.46	0.66a	0.26	0.56a	0.10
	6			0.04a	0.04	0.18a	0.20	0.00a	0.00			0.40a	0.16	0.70a	0.31	0.27a	0.17
Ethyl hexanoate (22 μg/l[b])	2	0.00a	0.00	0.00a	0.00	0.07b	0.04	0.00a	0.00	0.00a	0.00	0.00a	0.00	0.00a	0.00	0.00a	0.00
	6			0.02a	0.03	0.00a	0.00	0.00a	0.00			0.00a	0.00	0.02a	0.01	0.01a	0.01
Hexyl acetate (2[a])	2	10.77a	2.66	3.93a	1.30	6.78a	2.38	53.07b	6.50	10.95b	2.32	4.07a	1.61	4.90a	1.58	6.51a	0.89
	6			1.16a	0.53	2.37a	1.80	14.03b	3.29			2.88a	1.19	4.05a	1.50	3.09a	1.13
Propyl hexanoate (nf)	2	0.02b	0.01	0.00a	0.00	0.01a	0.00	0.00a	0.00	0.10a	0.02	0.08a	0.04	0.07a	0.03	0.06a	0.01
	6			0.00a	0.00	0.00a	0.00	0.00a	0.00			0.06a	0.03	0.11a	0.03	0.04a	0.05
Hexyl propanoate (8[b])	2	0.35b	0.05	0.02a	0.01	0.04a	0.02	0.09a	0.03	0.35b	0.11	0.15a	0.07	0.16a	0.05	0.14a	0.01
	6			0.01a	0.01	0.03a	0.04	0.06a	0.04			0.11a	0.04	0.13a	0.04	0.13a	0.06
	2	2.20b	1.01	0.21a	0.07	0.58a	0.14	0.13a	0.06	2.44a	0.50	1.88a	0.57	1.62a	0.35	1.88a	0.13

Butyl hexanoate (700[a])	6			0.32a	0.19	0.86a	0.72	0.09a	0.04			3.51a	0.55	6.38b	1.13	2.45a	0.92
Hexyl butanoate (250[b])	2	1.33b	0.53	0.23a	0.04	0.37a	0.08	0.66a	0.15	1.13a	0.53	0.66a	0.18	0.57a	0.12	0.64a	0.08
	6			0.24a	0.08	0.36a	0.21	0.53a	0.18			1.37a	0.43	1.94a	0.44	1.31a	0.27
Hexyl hexanoate (6400[b])	2	1.07b	0.62	0.28a	0.08	0.33a	0.13	0.21a	0.12	1.01a	0.63	1.27a	0.46	0.88a	0.07	1.40a	0.20
	6			2.05a	0.74	3.64ab	1.04	6.09b	1.58			14.41a	3.48	21.56a	4.94	17.38a	3.42
Butyl octanoate (nf)	2	0.27b	0.13	0.04a	0.01	0.09a	0.04	0.01a	0.01	0.37a	0.17	0.25a	0.10	0.23a	0.09	0.26a	0.02
	6			0.02a	0.02	0.09a	0.07	0.01	0.01			0.22a	0.04	0.42b	0.13	0.16a	0.07
**Branched esters**																	
2-Methylpropyl acetate (66[a])	2	0.00a	0.00	0.00a	0.00	0.11b	0.04	0.00a	0.00	0.00b	0.00	0.26a	0.13	0.26a	0.04	0.33a	0.04
	6			0.00a	0.00	0.00a	0.00	0.00a	0.00			0.00a	0.00	0.00a	0.00	0.00a	0.00
Ethyl 2-methylbutanoate (0.06[a])	2	0.00a	0.00	0.00a	0.00	0.02b	0.01	0.00a	0.00	0.01b	0.00	0.00a	0.00	0.00a	0.00	0.00a	0.00
	6			0.00a	0.00	0.00a	0.00	0.00a	0.00			0.00a	0.00	0.00a	0.00	0.00a	0.00
2-Methylbutyl acetate (11[a])	2	2.03a	0.26	2.91a	0.42	5.41b	0.91	4.52b	0.78	5.08a	0.88	6.10ab	1.54	7.10b	1.19	9.07ab	1.74
	6			6.38a	3.04	8.18a	2.79	10.15a	2.48			6.87a	3.19	8.27a	2.92	6.21a	1.15
Butyl 2-methylbutanoate (17[e])	2	0.70a	0.19	0.04a	0.02	0.13b	0.03	0.04b	0.01	2.88b	0.59	0.59a	0.49	0.63a	0.29	0.63a	0.16
	6			0.01a	0.02	0.08a	0.12	0.00a	0.00			0.28a	0.12	0.52a	0.32	0.17a	0.10
2-Methylbutyl butanoate (17[e])	2	0.00a	0.00	0.00a	0.00	0.00a	0.00	0.00a	0.00	0.00a	0.00	0.00a	0.00	0.00a	0.00	0.00a	0.00
	6			0.04a	0.01	0.03a	0.02	0.00b	0.00			0.12a	0.01	0.16b	0.02	0.13a	0.01
Amyl 2-methylbutanoate (nf)	2	0.02b	0.00	0.00a	0.00	0.00a	0.00	0.00a	0.00	0.09b	0.03	0.04a	0.02	0.03a	0.01	0.03a	0.00
	6			0.00a	0.00	0.00a	0.00	0.00a	0.00			0.03a	0.01	0.04a	0.02	0.01a	0.01
5-Hexenyl acetate (7[a])	2	0.06ab	0.02	0.03b	0.00	0.06b	0.02	0.06b	0.01	0.07b	0.01	0.06a	0.01	0.07a	0.01	0.09a	0.01
	6			0.02a	0.00	0.03a	0.02	0.08b	0.01			0.05a	0.02	0.09a	0.03	0.05a	0.02
Hexyl 2-methylbutanoate (22[d])	2	1.31b	0.29	0.32a	0.08	0.35a	0.07	0.35a	0.05	2.72b	0.87	1.02a	0.35	0.89a	0.24	1.19a	0.16
	6			0.36a	0.06	0.42a	0.18	1.06a	0.30			1.66a	0.71	2.04a	0.78	1.41a	0.33

**Alcohols**																	
Ethanol (100000[a])	2	0.21a	0.08	0.30a	0.08	3.04b	1.32	0.42a	0.19	0.35a	0.32	0.14a	0.05	0.29a	0.15	0.22a	0.05
	6			0.43a	0.15	0.45a	0.06	0.55a	0.05			0.54a	0.06	0.85b	0.18	0.52a	0.02
2-Pentanol (nf)	2	0.00a	0.00	0.00a	0.00	0.00a	0.00	0.80b	0.70	0.00a	0.00	0.00a	0.00	1.07a	1.24	0.03a	0.00
	6			0.00a	0.00	0.61a	1.12	1.21a	0.26			0.00a	0.00	0.00a	0.00	0.77b	0.11
1-Butanol (500[a])	2	0.00a	0.00	0.55a	0.15	3.51b	1.34	2.86a	0.27	0.00a	0.00	0.00a	0.00	2.35b	1.54	1.64b	0.68
	6			0.46	0.24	1.05a	1.02	0.32a	0.10			0.65a	0.36	1.10a	0.81	0.52a	0.20
2-Methyl-1-butanol (250[a])	2	0.07a	0.00	0.16ab	0.03	0.32c	0.11	0.29bc	0.01	0.23a	0.02	0.25a	0.10	0.33a	0.07	0.28a	0.04
	6			0.57a	0.19	0.55a	0.15	1.10b	0.31			0.35a	0.14	0.49a	0.26	0.43a	0.08
1-Hexanol (500[a])	2	0.92a	0.11	0.57a	0.16	1.04a	0.44	4.53b	1.00	0.67a	0.17	0.36a	0.11	0.47a	0.14	0.59a	0.16
	6			0.50a	0.16	0.51a	0.17	4.49b	0.79			0.47a	0.12	0.57a	0.15	1.22a	0.08
**Other VOCs**																	
2-Pentanone (2300[c])	2	0.00a	0.00	0.00a	0.00	0.00b	0.00	0.09b	0.03	0.00a	0.00	0.00a	0.00	0.00a	0.00	0.03b	0.01
	6			0.00a	0.00	0.00a	0.00	1.06b	0.46			0.00a	0.00	0.00a	0.00	0.21b	0.10
Hexanal (5[a])	2	0.00a	0.00	0.00a	0.00	0.00a	0.00	0.05b	0.00	0.00a	0.00	0.00a	0.00	0.00a	0.00	0.00a	0.00
	6			0.17a	0.10	0.08a	0.03	0.27b	0.08			0.06a	0.03	0.06a	0.01	0.06a	0.01
6-Methyl-5-heptene-2-one (50[a])	2	0.01a	0.00	0.01a	0.00	0.01a	0.00	0.01a	0.01	0.01a	0.00	0.03ab	0.01	0.03ab	0.00	0.04b	0.01
	6			0.16a	0.03	0.20a	0.05	0.16a	0.03			0.08a	0.03	0.14b	0.01	0.19c	0.01
Benzaldehyde (150[c])	2	0.02a	0.01	0.02a	0.00	0.02a	0.01	0.02a	0.01	0.02a	0.01	0.02a	0.00	0.03a	0.02	0.03a	0.03
	6			0.06a	0.00	0.06a	0.00	0.07b	0.00			0.04a	0.00	0.13a	0.16	0.05a	0.01
Estragole (16[b])	2	0.22a	0.11	0.38a	0.08	0.45b	0.14	0.19a	0.04	0.59a	0.32	0.78a	0.28	0.76a	0.18	0.61a	0.06
	6			0.98a	0.32	0.79a	0.11	0.91a	0.33			0.61a	0.36	0.64a	0.38	0.33a	0.13
α-Farnesene (nf)	2	0.73ab	0.44	1.14a	0.16	0.98a	0.20	0.25b	0.23	1.84a	0.49	2.32a	0.49	1.79a	0.11	2.07a	0.39
	6			2.92a	0.44	3.78a	0.29	3.52b	0.84			3.54a	1.20	3.51a	0.40	4.15a	0.09

avg: average; sd: standard deviation

Means followed by equal letters, in the same month of storage and day of shelf life, do not differ by the Tukey test, at 5 % probability.

* Odor Threshold (OT) (µg kg-1), VOC: volatile compund

[a] [31]; [b][36]; [c][48]; [d][4]; [e] [49]; nf: not found
